# ABO Blood Types and Mortality in Patients Undergoing Hemodialysis

**DOI:** 10.1016/j.ekir.2026.106558

**Published:** 2026-04-22

**Authors:** Masafumi Kurajoh, Tetsuo Shoji, Shinya Nakatani, Yuki Nagata, Hisako Fujii, Yasuo Imanishi, Masanori Emoto, Tomoaki Morioka

**Affiliations:** 1 Department of Metabolism, Endocrinology and Molecular Medicine, Osaka Metropolitan University Graduate School of Medicine, Osaka, Japan; 2 Department of Vascular Medicine, Osaka Metropolitan University Graduate School of Medicine, Osaka, Japan; 3 Vascular Science Center for Translational Research, Osaka Metropolitan University Graduate School of Medicine, Osaka, Japan; 4 Clinical Research Center, Aijinkai Inoue Hospital, Suita, Japan; 5 Department of Nephrology, Osaka Metropolitan University Graduate School of Medicine, Osaka, Japan; 6 Department of Health and Medical Innovation, Osaka Metropolitan University Graduate School of Medicine, Osaka, Japan

**Keywords:** ABO blood type, all-cause mortality, blood type A, cardiovascular mortality, hemodialysis

## Abstract

**Introduction:**

ABO blood types have been linked to cardiovascular and noncardiovascular mortality in nondialysis populations, but whether these associations extend to patients undergoing hemodialysis remains unclear. This study aimed to examine the associations between ABO blood types and all-cause, cardiovascular, and noncardiovascular mortality in these patients.

**Methods:**

This multicenter prospective cohort study included 1671 patients undergoing hemodialysis from 17 dialysis facilities in Japan, followed for up to 5 years. ABO blood type was obtained from medical records. Outcomes were all-cause, cardiovascular, and noncardiovascular mortality.

**Results:**

ABO blood type distribution was A: 650 (38.9%), B: 358 (21.4%), AB: 176 (10.5%), and O: 487 (29.1%). During 5 years of follow-up, 464 deaths occurred, including 278 cardiovascular and 186 noncardiovascular. In multivariable Cox regression models, blood type A, but not blood types B or AB, was associated with a significantly lower risk of all-cause mortality compared with blood type O (hazard ratio [HR]: 0.780; 95% confidence interval [CI]: 0.619–0.981; *P* = 0.034). In Fine–Gray subdistribution models, blood type A, but not blood types B or AB, was also associated with a lower risk of cardiovascular mortality. Exploratory analyses comparing blood type A with non-A blood types for cardiovascular mortality yielded similar results, with consistent findings across subgroups and sensitivity analyses. In contrast, no significant association was observed between ABO blood type and noncardiovascular mortality.

**Conclusion:**

ABO blood type may be associated with all-cause and cardiovascular mortality in patients undergoing hemodialysis, with lower risks observed in those with blood type A.

## Graphical abstract

**Figure:**
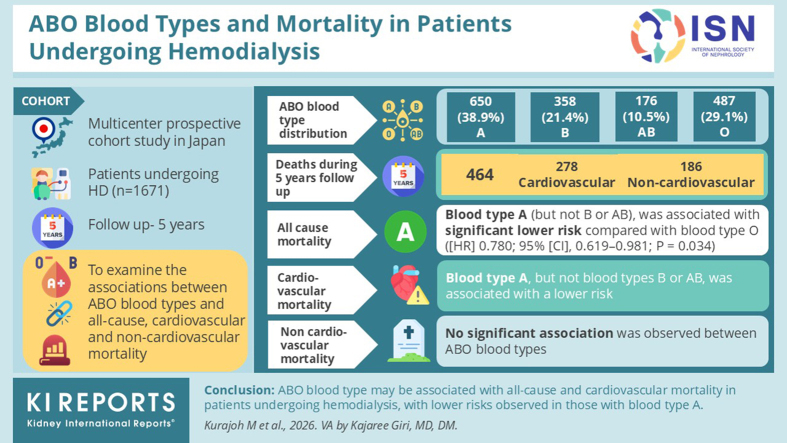

Patients undergoing hemodialysis face a substantially higher risk of both cardiovascular and noncardiovascular mortality than the general population.^1^ Despite substantial advances in dialysis care, including improvements in dialyzer membranes, fluid management, and pharmacotherapy, overall survival remains suboptimal.^2^^,^^3^ Although established risk factors such as age, diabetes, smoking, malnutrition, inflammation, and previous cardiovascular disease are well recognized,^4^, ^5^, ^6^ there remains a need for simple, noninvasive, and genetically determined markers that provide complementary information for risk assessment in this high-risk population. Genetically determined traits, such as the ABO blood group, may contribute to this role.

ABO blood group antigens are not only fundamental in transfusion medicine but have also been implicated in the pathophysiology of various diseases. In nondialysis populations, previous studies have reported associations of O blood groups with a reduced risk of cardiovascular events, including coronary artery disease , stroke, and peripheral artery disease ,^7^^,^^8^ as well as cardiovascular mortality.^9^ In addition, ABO blood types have been linked to susceptibility to noncardiovascular conditions, such as certain cancers and infectious diseases,^10^ and to noncardiovascular mortality related to these disorders.^11^^,^^12^ Collectively, these findings suggest that ABO blood type may influence mortality in nondialysis populations. However, whether such associations extend to patients undergoing hemodialysis remains unclear and warrants further investigation.

Based on these observations, this study aimed to investigate the associations between ABO blood types and mortality in patients undergoing hemodialysis. Specifically, we evaluated the associations with all-cause, cardiovascular, and noncardiovascular mortality in our multicenter prospective cohort of more than 1600 patients. Clarifying these associations may provide complementary insights into the role of genetically determined traits in mortality risk in this high-risk population.

## Methods

### Study Design and Participants

The Osaka dialysis complication study (ODCS) was a prospective cohort study of prevalent patients undergoing maintenance hemodialysis. Details of the study protocol have been described elsewhere.^13^ A total of 1696 patients from 17 dialysis facilities in Osaka Prefecture, Japan, were enrolled and followed from 2012 to 2017 for all-cause, cardiovascular, and noncardiovascular mortality. Annual case report forms which were filled by attending nephrologists were sent to the data center at Osaka City University School of Medicine and compiled after data cleaning. Some findings from the ODCS have been reported previously.^13^, ^14^, ^15^, ^16^, ^17^, ^18^ The present analysis examined the associations of ABO blood types with these mortality outcomes. We excluded participants with missing data for key baseline variables, including ABO blood type.

### Ethics Statement

The ODCS was conducted in accordance with the Declaration of Helsinki. The study protocol was approved by the ethics committee of Osaka City University Graduate School of Medicine (approval No. 2219) and registered in the UMIN Clinical Trials Registry (UMIN000007470). The revised protocol was subsequently approved by the ethics committee of Osaka Metropolitan University Graduate School of Medicine (approval No. 2021-029). Written informed consent was obtained from all participants before enrollment.

### ABO Blood Types

The ABO blood types (A, B, AB, or O) of each participant were obtained from medical records, in which blood typing had been serologically determined by the agglutination test.

### Outcomes

The outcomes of interest were as follows: (i) all-cause mortality, (ii) cardiovascular mortality, and (iii) noncardiovascular mortality.

1. All-cause mortality was defined as death from any cause.
2. Cardiovascular mortality included deaths attributable to coronary artery disease , stroke, congestive heart failure, peripheral artery disease, aortic dissection, or sudden death.
3. Noncardiovascular mortality included all other causes, such as infection, malignancy, or trauma and fracture.

Sudden death was predefined as death occurring within 24 hours of onset in the absence of evidence of accident or crime. For deaths other than sudden death, causes of death were adjudicated based on clinical information from the final hospitalization preceding death.

### Other Variables

All demographic, clinical, laboratory, and medication variables were assessed at study baseline (2012). We collected demographic and clinical data, including age, sex, body mass index (BMI), dialysis vintage, dialysis adequacy, underlying kidney disease (specifically the presence or absence of diabetic kidney disease, hypertension, dyslipidemia, smoking status, history of cardiovascular disease events, and history of hospitalization for heart failure. Medication use at baseline included erythropoiesis-stimulating agents, oral and/or intravenous iron preparations, vitamin D receptor activators, antiplatelet agents, anticoagulants, β-blockers, antihypertensive agents (angiotensin-converting enzyme inhibitors and/or angiotensin II receptor blockers), and statins.

Laboratory parameters included hemoglobin, serum albumin, C-reactive protein , calcium, phosphate, intact parathyroid hormone, and alkaline phosphatase (ALP). Hypertension was defined as systolic blood pressure ≥ 140 mm Hg, diastolic blood pressure ≥ 90 mm Hg, and/or antihypertensive medication use.^19^ Dyslipidemia was defined as triglycerides ≥175 mg/dl, high-density lipoprotein cholesterol ≤ 40 mg/dl, non– high-density lipoprotein cholesterol ≥ 150 mg/dl, low-density lipoprotein cholesterol ≥120 mg/dl, and/or use of lipid-lowering agents.^20^

### Statistical Analysis

Participants with missing data for key baseline variables used in the primary analyses were excluded, and analyses were conducted using complete cases. Participants lost to follow-up because of relocation or transfer, those who switched to peritoneal dialysis, and those who underwent kidney transplantation were censored at the time of their last confirmed follow-up.

Clinical and demographic characteristics were presented as medians with interquartile ranges (IQRs) for continuous variables and as counts with percentages for categorical variables. Comparisons among the 4 ABO blood groups were made using the Kruskal–Wallis test for continuous variables and the chi-squared test for categorical variables.

The association between ABO blood types and all-cause mortality was evaluated using the Kaplan–Meier method and compared with the log-rank test. Cox proportional hazards regression models were constructed using 4-group models with blood type O as the reference category. Multivariable models were adjusted for potential confounders as follows: (i) major demographic factors (age, sex, dialysis vintage, diabetic kidney disease, and history of cardiovascular disease events); (ii) traditional risk factors (current smoking, hypertension, and dyslipidemia); (iii) factors related to renal anemia (hemoglobin level, use of erythropoiesis-stimulating agents, and use of iron preparations); (iv) factors related to chronic kidney disease (CKD)–mineral and bone disorder (serum calcium, phosphate, intact parathyroid hormone, and use of vitamin D receptor activators); and (v) factors related to inflammation and wasting (BMI, serum albumin, and C-reactive protein, log-transformed). The proportional hazards assumption for Cox models was assessed using Schoenfeld residuals.

For cause-specific mortality, competing risk analyses were performed for cardiovascular and noncardiovascular death. Cumulative incidence functions were estimated and compared using Gray’s test. Adjusted associations were evaluated using Fine–Gray subdistribution hazard models with the same covariates as in the Cox models. In assessing cardiovascular mortality, noncardiovascular death was treated as a competing event, and vice versa. Proportionality in Fine–Gray models was assessed using time-by-covariate interaction terms and visual inspection of cumulative incidence curves.

In the primary competing risk analyses, ABO blood type was modeled using 4 categories with blood type O as the reference. Based on these results, additional analyses were conducted using a dichotomized comparison of blood type A versus non-A. Because this comparison was not prespecified in the original statistical analysis plan, no formal adjustment for multiple comparisons was applied, and the results were interpreted as exploratory.

Subgroup analyses for cardiovascular mortality using the A versus non-A classification were performed across clinically relevant categories. Continuous variables were dichotomized at the median, except for age (65 years) and dialysis vintage (5 years). Interactions between blood type A (vs. non-A) and subgroup variables were assessed by including interaction terms in the models.

Sensitivity analyses for cardiovascular mortality were conducted using the A versus non-A classification. These included additional adjustment for the following: (i) antiplatelet agents, (ii) anticoagulants, (iii) β-blockers, antihypertensive agents, and statins, (iv) ALP, (v) history of hospitalization for heart failure, (vi) dialysis adequacy, and (vii) a propensity score–matched analysis. In the propensity score–matched analysis, patients were matched 1:1 using nearest-neighbor matching with a caliper width of 0.2. Adequate balance was defined as an absolute standardized difference < 0.05. Additional analyses were performed separating cardiovascular mortality into sudden and nonsudden cardiovascular death.

Additionally, incremental predictive performance for all-cause mortality was evaluated using Cox models, as established methods for incremental prediction are not well defined for competing risk models. Improvements in model discrimination after adding ABO blood type (modeled as 4 categories) to a baseline model were assessed using the C-statistic and likelihood ratio tests.

Statistical analyses were performed using the Statistical Package for the Social Sciences software package (version 27.0, IBM Corp, Armonk, NY) and the Easy-R (EZR) software package (version 1.66, Saitama Medical Center, Jichi Medical University, Saitama, Japan).^21^ Additional analyses were conducted using R scripts implemented within the EZR environment. All P values were 2-sided and considered statistical significance at *P* < 0.05.

## Results

### Selection of Patients

Of the 1696 participants, those with missing data on ABO blood type (*n* = 7), hypertension (*n* = 3), dyslipidemia (*n* = 4), or BMI (*n* = 11) were excluded. Finally, 1671 patients were included in the analysis (Supplementary Figure S1).

### Clinical Characteristics of Patients

Table 1 summarizes the baseline characteristics of the participants (*n* = 1671). The distribution of ABO blood types was as follows: (i) type A in 650 patients (38.9%), (ii) type B in 358 (21.4%), (iii) type AB in 176 (10.5%), and (iv) type O in 487 (29.1%). Significant differences were observed in age, ALP levels, and the use of anticoagulants and statins among the 4 groups, whereas no significant differences were observed in other clinical variables.

**Table 1:** Baseline clinical characteristics of patients according to ABO blood type (*N* = 1671)

Variables	Total (*N* = 1671)	A (*n* = 650)	B (*n* = 358)	AB (*n* = 176)	O (*n* = 487)	*P*-value
Age, yrs	68 (60–75)	66 (59–74)	68 (61–75)	70 (61–76)	68 (60–74)	0.016
Female sex, *n* (%)	619 (37.0%)	232 (35.7%)	128 (35.8%)	70 (39.8%)	189 (38.8%)	0.575
BMI, kg/m^2^	21.1 (19.0–23.6)	21.3 (19.0–23.6)	21.2 (18.9–23.6)	21.0 (19.3–23.8)	21.0 (18.8–23.4)	0.602
Current smoking, *n* (%)	258 (15.4%)	112 (17.2%)	53 (14.8%)	18 (10.2%)	75 (15.4%)	0.141
Underlying disease and comorbidities						
DKD, *n* (%)	683 (40.9%)	269 (41.4%)	144 (40.2%)	70 (39.8%)	200 (41.1%)	0.974
Hypertension, *n* (%)	1509 (90.3%)	592 (91.1%)	327 (91.3%)	157 (89.2%)	433 (88.9%)	0.525
Dyslipidemia, *n* (%)	1035 (61.9%)	414 (63.7%)	204 (57.0%)	110 (62.5%)	307 (63.0%)	0.186
Previous medical history						
CVD, *n* (%)	633 (37.9%)	246 (37.8%)	132 (36.9%)	66 (37.5%)	189 (38.8%)	0.953
Hospitalization for heart failure, *n* (%)	225 (13.5%)	86 (13.2%)	56 (15.6%)	18 (10.2%)	65 (13.3%)	0.388
Dialysis-related parameters						
Dialysis vintage, mos	64 (29–132)	64 (28–133)	66 (30–126)	60 (22–124)	64 (31–141)	0.341
Kt/V	1.38 (1.20–1.57)	1.38 (1.20–1.57)	1.38 (1.20–1.54)	1.40 (1.20–1.56)	1.38 (1.20–1.59)	0.964
Laboratory parameters						
Hemoglobin, g/dl	10.6 (10.0–11.3)	10.7 (10.0–11.3)	10.6 (9.9–11.4)	10.7 (9.9–11.4)	10.6 (10.0–11.2)	0.967
Albumin, g/dl	3.7 (3.5–3.9)	3.7 (3.5–3.9)	3.7 (3.5–3.9)	3.7 (3.5–3.9)	3.7 (3.5–3.9)	0.573
CRP, mg/dl	0.10 (0.05–0.32)	0.10 (0.05–0.3)	0.10 (0.05–0.33)	0.10 (0.05–0.28)	0.10 (0.05–0.35)	0.808
Calcium, mg/dl	8.9 (8.5–9.4)	8.9 (8.5–9.4)	8.9 (8.5–9.5)	9.1 (8.5–-9.6)	8.9 (8.4–9.4)	0.205
Phosphate, mg/dl	5.1 (4.3–5.9)	5.1 (4.3–5.8)	5.1 (4.3–6.0)	4.9 (4.1–5.8)	5.1 (4.3–6.1)	0.091
Intact PTH, pg/ml	114 (60–186)	118 (65–182)	115 (55–194)	105 (53–164)	114 (59–199)	0.238
ALP, U/l	227 (175–299)	188 (155–243)	250 (201–325)	206 (170–268)	270 (207–343)	< 0.001
Medications						
ESA, *n* (%)	1376 (82.3%)	538 (82.8%)	291 (81.3%)	147 (83.5%)	400 (82.1%)	0.912
Iron preparations, *n* (%)	466 (27.9%)	182 (28.0%)	99 (27.7%)	48 (27.3%)	137 (28.1%)	0.997
VDRA, *n* (%)	1143 (68.4%)	447 (68.8%)	255 (71.2%)	116 (65.9%)	325 (66.7%)	0.472
Antiplatelet agents, *n* (%)	769 (46.0%)	316 (48.6%)	151 (42.2%)	85 (48.3%)	217 (44.6%)	0.197
Anticoagulants, *n* (%)	133 (8.0%)	42 (6.5%)	38 (10.6%)	9 (5.1%)	44 (9.0%)	0.045
β-blockers, *n* (%)	589 (35.3%)	201 (30.9%)	100 (27.9%)	56 (31.8%)	132 (27.1%)	0.419
ACEIs and/or ARBs, *n* (%)	843 (50.4%)	350 (53.8%)	175 (48.9%)	83 (47.2%)	235 (48.3%)	0.168
Statins, *n* (%)	255 (15.3%)	125 (19.2%)	51 (14.2%)	17 (9.7%)	62 (12.7%)	0.002

ACEI, angiotensin-converting enzyme inhibitor; ALP, alkaline phosphatase; ARB, angiotensin II receptor blocker.

BMI, body mass index; CRP, C-reactive protein; CVD, cardiovascular disease; DKD, diabetic kidney disease; ESA, erythropoiesis-stimulating agents; PTH, parathyroid hormone; VDRA, vitamin D receptor activators.

Values are expressed as median (interquartile range) for continuous variables and as number (percentage) for categorical variables. *P*-values represent comparisons among the 4 blood type groups (A, B, AB, and O), calculated using the chi-square test for categorical variables and the Kruskal–Wallis test for continuous variables. Kt/V was available in 1590 of 1671 patients.

### Observed Outcomes During the Follow-Up Period

From 2012 to 2017, a total of 464 deaths occurred during a median follow-up of 1826 days (IQR: 884–1826). Of these, 278 deaths (59.9%) were classified as cardiovascular, including coronary artery disease (*n* = 44), stroke (*n* = 52), congestive heart failure (*n* = 38), peripheral artery disease (*n* = 20), aortic dissection (*n* = 1), and sudden death (*n* = 123). The remaining 186 deaths (40.1%) were noncardiovascular, comprising infection (*n* = 123), malignancy (*n* = 36), and trauma or fracture (*n* = 27).

### ABO Blood Type and All-Cause Mortality

**Figure 1:**
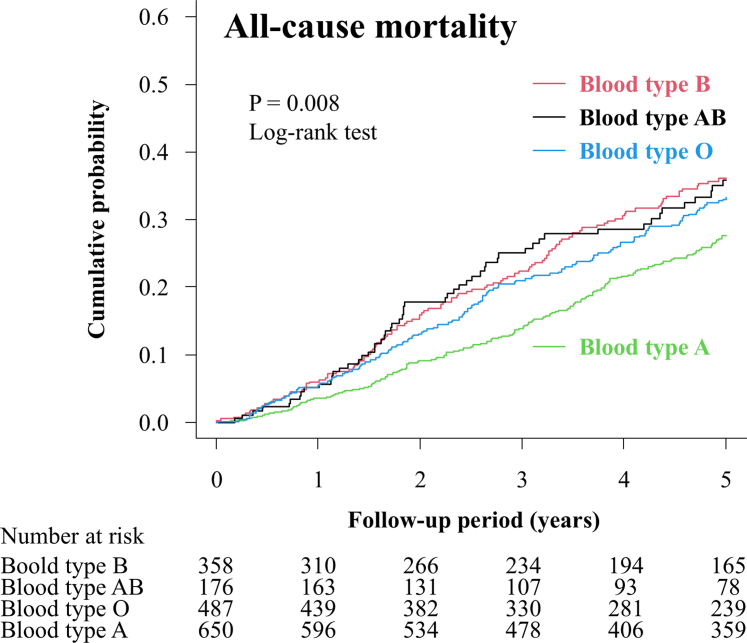
Kaplan–Meier survival curves for all-cause mortality according to ABO blood type. Kaplan–Meier survival curves showing the cumulative probability of all-cause mortality, stratified by ABO blood type. The *P*-value was calculated using the log-rank test.

Kaplan–Meier survival curves (Figure 1) demonstrated significant differences in all-cause mortality among the 4 ABO blood types (log-rank test, *P* = 0.008), with blood type A exhibiting the lowest risk.

To determine whether ABO blood type was associated with all-cause mortality independently of potential confounders, multivariable Cox proportional hazards regression analyses were conducted (Table 2). In these 4-group models with blood type O as the reference, blood type A was associated with a significantly lower risk of all-cause mortality (hazard ratio [HR]: 0.780; 95% confidence interval [CI]: 0.619–0.981; *P* = 0.034), whereas blood types B and AB were not significantly associated with mortality.

**Table 2:** Multivariable Cox proportional hazards model for all-cause mortality

Variables	HR (95% CI)	*P*-value
Age (per 1-yr higher)	1.058 (1.046–1.070)	< 0.001
Female (ref. male)	0.835 (0.684–1.021)	0.079
Dialysis vintage (per 1-mo higher)	1.001 (1.000–1.002)	0.291
DKD (ref. absence)	1.633 (1.339–1.990)	< 0.001
Previous CVD (ref. absence)	1.770 (1.462–2.143)	< 0.001
Current smoking (ref. absence)	1.150 (0.863–1.533)	0.339
Hypertension (ref. absence)	0.719 (0.529–0.978)	0.035
Dyslipidemia (ref. absence)	0.952 (0.782–1.159)	0.627
Hemoglobin (per 1-g/dl higher)	1.046 (0.959–1.140)	0.308
Use of ESA (ref. absence)	1.125 (0.871–1.453)	0.367
Use of iron preparations (ref. absence)	0.942 (0.764–1.161)	0.573
Calcium (per 1-mg/dl higher)	1.057 (0.920–1.214)	0.431
Phosphate (per 1-mg/dl higher)	0.987 (0.914–1.065)	0.728
Intact PTH (per 100-pg/ml higher)	1.044 (0.996–1.094)	0.071
Use of VDRA (ref. absence)	0.993 (0.811–1.216)	0.948
BMI (per 1-kg/m^2^ higher)	0.929 (0.901–0.959)	< 0.001
Albumin (per 1-g/dl higher)	0.429 (0.321–0.574)	< 0.001
Log CRP (per 1-unit higher)	1.314 (1.109–1.557)	0.002
Blood type A (ref. O)	0.780 (0.619–0.981)	0.034
Blood type B (ref. O)	1.108 (0.861–1.426)	0.424
Blood type AB (ref. O)	1.040 (0.758–1.428)	0.808

BMI, body mass index; CI, confidence interval; CRP, C-reactive protein; CVD, cardiovascular disease; DKD, diabetic kidney disease; ESA, erythropoiesis-stimulating agents; HR, hazard ratio; PTH, parathyroid hormone; VDRA, vitamin D receptor activators.

CRP values were log-transformed before inclusion in the multivariable model.

### ABO Blood Type and Cardiovascular Mortality

**Figure 2:**
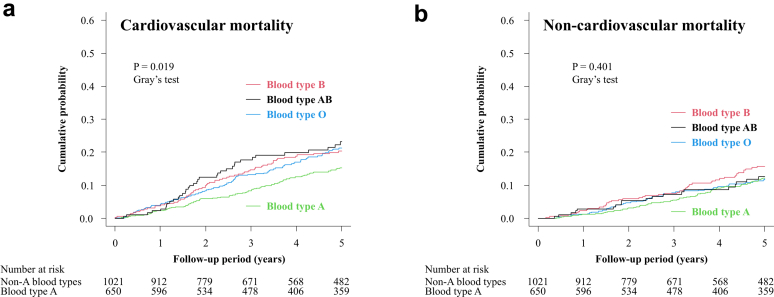
Cumulative incidence functions for cause-specific mortality according to ABO blood type. Cumulative incidence functions showing cardiovascular mortality (a) and non-cardiovascular mortality (b), stratified by ABO blood type. The *P*-value was calculated using Gray’s test.

The cumulative incidence of cardiovascular mortality differed significantly among the 4 ABO blood groups (Gray’s test, *P* = 0.019) (Figure 2A), with blood type A showing the lowest risk. To evaluate whether these differences were independent of potential confounders, Fine–Gray subdistribution hazard model analyses were performed (Table 3). In the primary 4-group analyses with blood type O as the reference, blood type A—but not blood types B or AB—was significantly associated with a lower risk of cardiovascular mortality (HR: 0.723; 95% CI: 0.535–0.978; *P* = 0.035).

**Table 3:** Fine–Gray subdistribution hazards model for cardiovascular and noncardiovascular mortality

Variables	Cardiovascular mortality		Noncardiovascular mortality	
	HR (95% CI)	*P*-value	HR (95% CI)	*P*-value
Age (per 1-yr higher)	1.055 (1.038–1.072)	< 0.001	1.041 (1.023–1.060)	< 0.001
Female (ref. male)	0.732 (0.562–0.955)	0.021	1.038 (0.756–1.425)	0.82
Dialysis vintage (per 1-mo higher)	1.000 (0.999–1.002)	0.72	1.001 (0.999–1.002)	0.35
DKD (ref. absence)	1.815 (1.390–2.370)	< 0.001	1.171 (0.853–1.608)	0.33
Previous CVD (ref. absence)	1.591 (1.240–2.040)	< 0.001	1.626 (1.190–2.223)	0.002
Current smoking (ref. absence)	0.954 (0.642–1.418)	0.81	1.401 (0.908–2.162)	0.13
Hypertension (ref. absence)	0.893 (0.559–1.426)	0.63	0.684 (0.426–1.099)	0.12
Dyslipidemia (ref. absence)	1.095 (0.847–1.417)	0.49	0.818 (0.603–1.110)	0.2
Hemoglobin (per 1-g/dl higher)	1.048 (0.934–1.175)	0.43	1.001 (0.873–1.146)	0.99
Use of ESA (ref. absence)	0.919 (0.674–1.253)	0.59	1.311 (0.835–2.058)	0.24
Use of iron preparations (ref. absence)	1.087 (0.837–1.412)	0.53	0.844 (0.596–1.197)	0.34
Calcium (per 1-mg/dl higher)	1.088 (0.913–1.296)	0.35	1.009 (0.808–1.259)	0.94
Phosphate (per 1-mg/dl higher)	0.975 (0.877–1.084)	0.64	1.033 (0.919–1.162)	0.59
Intact PTH (per 100-pg/ml higher)	1.005 (0.916–1.102)	0.92	1.071 (1.023–1.121)	0.003
Use of VDRA (ref. absence)	1.219 (0.929–1.599)	0.15	0.749 (0.539–1.041)	0.085
BMI (per 1-kg/m^2^ higher)	0.939 (0.900–0.979)	0.003	0.955 (0.908–1.003)	0.068
Albumin (per 1-g/dl higher)	0.569 (0.374–0.868)	0.009	0.522 (0.341–0.801)	0.003
Log CRP (per 1-unit higher)	1.167 (0.915–1.488)	0.21	1.358 (1.046–1.763)	0.021
Blood type A (ref. O)	0.723 (0.535–0.978)	0.035	1.064 (0.735–1.540)	0.74
Blood type B (ref. O)	0.929 (0.656–1.315)	0.68	1.385 (0.921–2.082)	0.12
Blood type AB (ref. O)	1.068 (0.719–1.585)	0.74	1.024 (0.590–1.778)	0.93

BMI, body mass index; CI, confidence interval; CRP, C-reactive protein; CVD, cardiovascular disease; DKD, diabetic kidney disease; ESA, erythropoiesis-stimulating agents; HR, hazard ratio; PTH, parathyroid hormone; VDRA, vitamin D receptor activators.

Note: CRP values were log-transformed before inclusion in the multivariable model.

**Figure 3:**
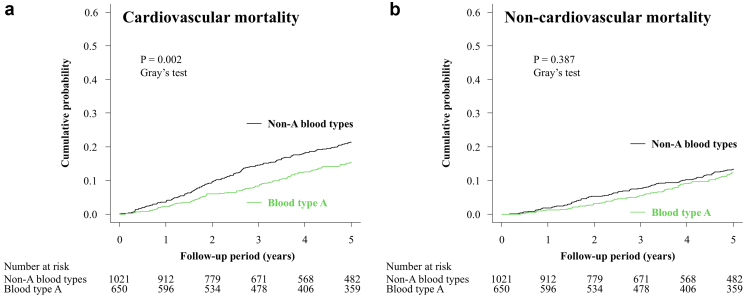
Cumulative incidence functions for cause-specific mortality according to blood type A versus non-A. Cumulative incidence functions showing cardiovascular mortality (a) and non-cardiovascular mortality (b), stratified by blood type A versus non-A. The *P* values were calculated using Gray’s test.

**Figure 4:**
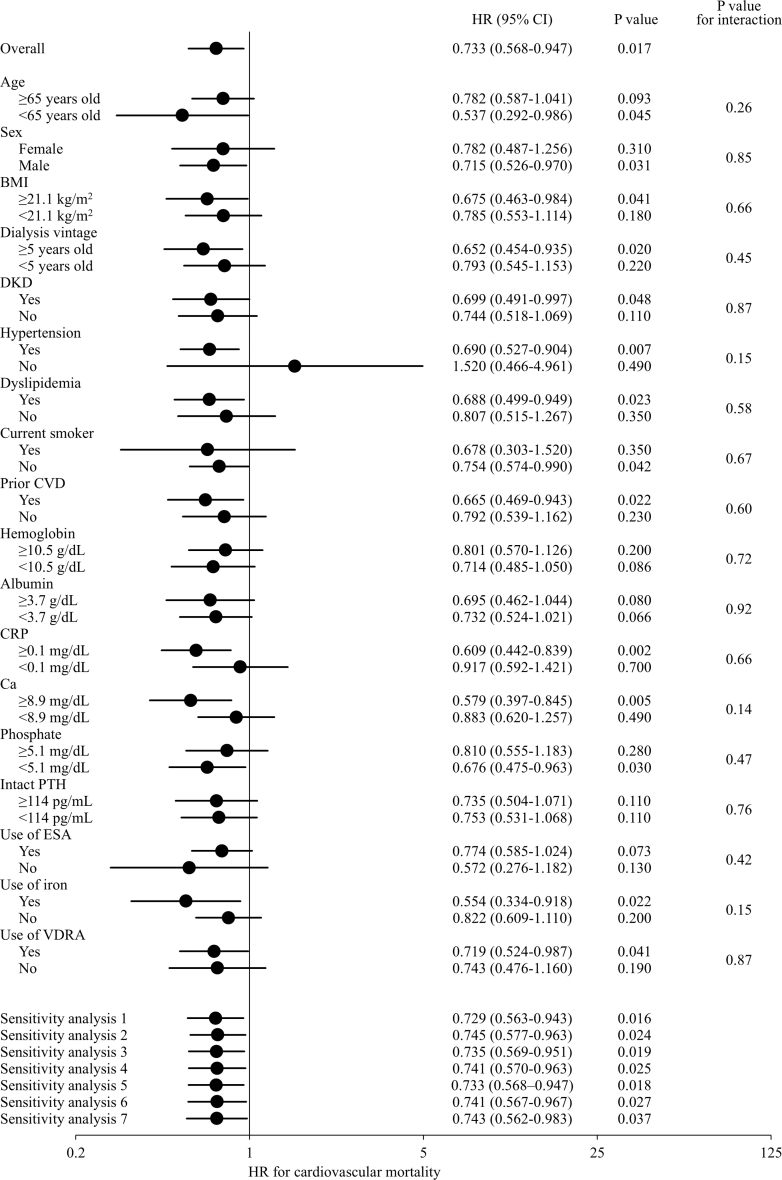
Subgroup and sensitivity analyses for the association between blood type A versus non-A and cardiovascular mortality. Forest plot showing HRs and 95% CIs for the association between blood type A versus non-A and cardiovascular mortality. Subgroup analyses were performed across clinically relevant categories. Seven sensitivity analyses were conducted: 1. additional adjustment for antiplatelet agents, 2. additional adjustment for anticoagulants, 3. additional adjustment for β-blockers, antihypertensive agents, and statins, 4. additional adjustment for serum ALP levels, 5. additional adjustment for history of hospitalization for heart failure, 6. additional adjustment for dialysis adequacy , and 7. propensity score–matched analysis. ALP, alkaline phosphatase; BMI, body mass index; CI, confidence interval; CVD, cardiovascular disease; CRP, C-reactive protein; DKD, diabetic kidney disease; ESA, erythropoiesis-stimulating agents; HR, hazard ratio; PTH, parathyroid hormone; VDRA, vitamin D receptor activators.

In secondary exploratory analyses using a dichotomized comparison, cumulative incidence functions showed lower cardiovascular mortality in patients with blood type A than in those with non-A blood types (Figure 3A; Gray’s test, *P* = 0.002). In Fine–Gray subdistribution hazard models, blood type A was significantly associated with a lower risk of cardiovascular mortality (HR: 0.733; 95% CI: 0.568–0.947; *P* = 0.017) (Table 4). Subgroup analyses showed no significant heterogeneity in the association between blood type A and cardiovascular mortality across clinically relevant subgroups (Figure 4; all *P* for interaction > 0.05). The association remained robust across sensitivity analyses, including additional adjustment for antiplatelet agents, anticoagulants, β-blockers, antihypertensive agents (angiotensin-converting enzyme inhibitors and/or angiotensin II receptor blockers), statins, ALP, history of hospitalization for heart failure, and dialysis adequacy (Figure 4). Additional analyses separating cardiovascular mortality into sudden and nonsudden cardiovascular death yielded similar results (Supplementary Table S1). In the propensity score–matched analysis, baseline clinical characteristics were well balanced between patients with blood type A and those with non-A blood types (all absolute standardized difference < 0.05; Supplementary Table S2), and the results were consistent with the primary findings (Figure 4).

**Table 4:** Fine–Gray subdistribution hazard model for cardiovascular and noncardiovascular mortality including ABO blood type (A vs. nonA)

Variables	Cardiovascular mortality		Noncardiovascular mortality	
	HR (95% CI)	*P*-value	HR (95% CI)	*P*-value
Age (per 1-yr higher)	1.055 (1.039–1.072)	< 0.001	1.041 (1.023–1.060)	< 0.001
Female (ref. male)	0.735 (0.564–0.957)	0.022	1.033 (0.752–1.418)	0.84
Dialysis vintage (per 1-mo higher)	1.000 (0.999–1.002)	0.72	1.001 (0.999–1.002)	0.35
DKD (ref. absence)	1.816 (1.392–2.370)	< 0.001	1.166 (0.849–1.601)	0.34
Previous CVD (ref. absence)	1.588 (1.238–2.037)	< 0.001	1.634 (1.196–2.232)	0.002
Current smoking (ref. absence)	0.956 (0.643–1.420)	0.82	1.392 (0.904–2.143)	0.13
Hypertension (ref. absence)	0.884 (0.557–1.404)	0.6	0.687 (0.430–1.099)	0.12
Dyslipidemia (ref. absence)	1.099 (0.850–1.421)	0.47	0.805 (0.594–1.090)	0.16
Hemoglobin (per 1-g/dL higher)	1.046 (0.933–1.173)	0.44	1.002 (0.874–1.148)	0.98
Use of ESA (ref. absence)	0.922 (0.677–1.257)	0.61	1.309 (0.834–2.057)	0.24
Use of iron preparations (ref. absence)	1.087 (0.836–1.412)	0.53	0.835 (0.589–1.185)	0.31
Calcium (per 1-mg/dL higher)	1.086 (0.911–1.293)	0.36	1.015 (0.813–1.269)	0.89
Phosphate (per 1-mg/dL higher)	0.974 (0.876–1.083)	0.63	1.032 (0.917–1.160)	0.6
Intact PTH (per 100-pg/mL higher)	1.005 (0.915–1.103)	0.92	1.070 (1.022–1.120)	0.004
Use of VDRA (ref. absence)	1.209 (0.925–1.581)	0.16	0.766 (0.551–1.066)	0.11
BMI (per 1-kg/m^2^ higher)	0.939 (0.901–0.979)	0.003	0.956 (0.909–1.006)	0.083
Albumin (per 1-g/dL higher)	0.579 (0.383–0.875)	0.010	0.520 (0.340–0.797)	0.003
Log CRP (per 1-unit higher)	1.168 (0.917–1.487)	0.21	1.367 (1.053–1.775)	0.019
Blood type A (ref. nonA)	0.733 (0.568–0.947)	0.017	0.940 (0.696–1.269)	0.69

BMI, body mass index; CI, confidence interval; CRP, C-reactive protein; CVD, cardiovascular disease; DKD, diabetic kidney disease; ESA, erythropoiesis-stimulating agents; HR, hazard ratio; PTH, parathyroid hormone; VDRA, vitamin D receptor activators.

CRP values were log-transformed before inclusion in the multivariable model.

### ABO Blood Type and Noncardiovascular Mortality

In contrast, cumulative incidence functions showed no significant differences in noncardiovascular mortality among the 4 ABO blood groups (Gray’s test, *P* = 0.401) (Figure 2). Consistently, Fine–Gray subdistribution hazard model analyses showed no significant association between ABO blood type and noncardiovascular mortality (Table 3). Similar nonsignificant findings were also observed in additional analyses using a dichotomized comparison of blood type A versus non-A, both in cumulative incidence functions (Figure 3B) and Fine–Gray subdistribution hazard models (Table 4).

## Discussion

In this multicenter prospective cohort of patients undergoing hemodialysis, ABO blood type was associated with all-cause and cardiovascular mortality, with a lower risk observed in blood type A, but not in blood types B or AB. Exploratory analyses comparing blood type A with non-A blood types for cardiovascular mortality yielded similar results, with consistent findings across subgroups and sensitivity analyses. In contrast, no significant association was observed between ABO blood type and noncardiovascular mortality. Taken together, these findings suggest that ABO blood type may be associated with cardiovascular mortality in patients undergoing hemodialysis, with lower risks observed in those with blood type A.

Evidence on the relationship between ABO blood type and mortality in end-stage kidney disease is limited and heterogeneous. A Canadian registry study (1981–1986) retrospectively analyzed patients with end-stage kidney disease, including those on hemodialysis, peritoneal dialysis, and kidney transplant recipients, and reported slightly lower mortality in blood type AB.^22^ However, its retrospective design and heterogeneous population limit the generalizability of these findings, particularly to patients undergoing hemodialysis. More recently, a Japanese single-center study of 365 patients undergoing dialysis found that blood type A was associated with a lower risk of a composite end point of cardio-cerebrovascular events and cardio-cerebrovascular death.^23^ Although directionally consistent with our findings, that single-center study was limited by its small sample and few events (73 events) and by the use of a cardio-cerebrovascular composite end point. Furthermore, it did not distinguish cardiovascular from noncardiovascular mortality and did not report multivariable regression analyses for all-cause mortality, despite a trend toward lower all-cause mortality (62 events) in blood type A. In contrast, in our multicenter prospective cohort of 1671 patients of hemodialysis, ABO blood type was associated with all-cause mortality (464 events) and cardiovascular mortality (278 events), but not noncardiovascular mortality (186 events). In particular, blood type A was associated with a lower risk of cardiovascular mortality. These associations were consistent across clinically relevant subgroups and multiple sensitivity analyses.

Unexpectedly, our study observed that blood type A, rather than blood type O, was associated with a lower risk of cardiovascular mortality in patients undergoing hemodialysis. In nondialysis populations, blood type O has consistently been linked to reduced cardiovascular mortality,^9^^,^^24^ partly because of lower plasma von Willebrand factor (VWF) and factor VIII levels,^25^ as higher levels are associated with worse prognosis.^26^^,^^27^ Even among patients undergoing hemodialysis, VWF and factor VIII levels have been reported to be lower in blood type O,^28^ and elevated VWF levels have been associated with higher mortality.^29^ Therefore, differences in VWF and factor VIII are unlikely to fully account for the lower cardiovascular mortality observed in patients with blood type A. To further assess antithrombotic pathways potentially contributing to this association, we performed a sensitivity analysis adjusting for antiplatelet agents and oral anticoagulants. The results remained unchanged, indicating that the lower cardiovascular mortality in patients with blood type A is unlikely to be explained by antithrombotic mechanisms. However, VWF and factor VIII were not measured in this study, and therefore the potential contribution of these pathways cannot be excluded.

We also considered the potential role of serum ALP, because previous studies have linked ABO blood type to ALP levels. At the baseline of this cohort (2012), ALP was measured using the Japan Society of Clinical Chemistry method in Japan. Previous studies have shown that ALP levels measured by this method are lower among individuals with blood type A in both nondialysis and dialysis populations,^30^^,^^31^ and this trend was also observed in our study (Table 1). Since elevated ALP has been reported to predict all-cause and, in some studies, cardiovascular mortality among patients of hemodialysis,^32^,^33^ we conducted a sensitivity analysis additionally adjusting for ALP. Results remained unchanged, suggesting that differences in ALP are unlikely to fully explain the lower mortality observed in patients with blood type A.

Beyond these established biochemical pathways, 1 possible explanation is that the unique pathophysiological environment of maintenance hemodialysis modifies the impact of ABO blood type on cardiovascular outcomes. Patients undergoing hemodialysis are characterized by chronic inflammation, vascular calcification, a uremic milieu, and repeated blood–membrane interactions.^34^, ^35^, ^36^, ^37^ These dialysis-specific features may contribute to cardiovascular risk patterns distinct from those in the general population, often described as reverse epidemiology.^38^^,^^39^ However, these mechanisms were not directly measured in this study and therefore remain speculative. Thus, our findings should be interpreted as hypothesis-generating rather than mechanistic.

Geographic and ethnic differences in ABO blood type distribution may also influence the observed associations. In Japan, type A is the most common, whereas type O predominates in many other countries.^40^^,^^41^ In our ODCS cohort, the distribution of ABO blood types (A: 38.9%, B: 21.4%, AB: 10.5%, and O: 29.1%) closely matched that of the general Japanese population (approximately 40%, 20%, 10%, and 30%, respectively),^41^ suggesting no population-specific selection bias. The reasons for such regional differences remain uncertain but may involve multiple factors, including founder effects, historical migration patterns, and differential susceptibility to infections, although natural selection driven by region-specific environmental or evolutionary pressures may also have contributed.^42^^,^^43^ Thus, the observed association between blood type A and cardiovascular mortality may, in part, reflect environmental and epidemiologic conditions unique to Japan. Further studies in diverse populations will be necessary to better understand the underlying mechanisms and assess the generalizability of these findings.

In additional analyses evaluating the incremental predictive performance for all-cause mortality, the addition of ABO blood type to a baseline Cox model resulted in a statistically significant improvement in model fit (*P* = 0.023); however, the increase in the C-statistic was modest (ΔC = 0.0025; Supplementary Table S3). These findings suggest that, although ABO blood type provides statistically significant prognostic information, its incremental value for individualized clinical risk prediction appears limited. Accordingly, our findings should not be interpreted as supporting changes in current cardiovascular prevention strategies in patients undergoing hemodialysis. Nevertheless, they may provide novel biological insights into mechanisms underlying cardiovascular mortality risk.

In contrast to its clear association with cardiovascular mortality, we found no significant relationship between ABO blood type and noncardiovascular mortality in patients undergoing hemodialysis. Notably, findings from nondialysis populations are inconsistent. For example, blood type O has been linked to higher mortality in sepsis, trauma, and gastrointestinal bleeding, but lower mortality in acute respiratory distress syndrome.^12^^,^^44^^,^^45^ Other reports show variable associations, including links between blood type A and increased COVID-19-related mortality and between blood type B and reduced sepsis-related mortality.^11^^,^^46^ Conversely, some studies have found no associations, such as with cancer-related mortality or in surgical and critical care settings.^9^^,^^47^^,^^48^ Overall, associations between ABO blood type and noncardiovascular mortality remain uncertain, as most studies focus on specific causes of death or selected populations. Furthermore, to date, no study has systematically evaluated this association in patients undergoing hemodialysis. To our knowledge, our study is the first to systematically evaluate these relationships in a relatively large hemodialysis cohort, indicating that ABO blood type has limited relevance to noncardiovascular mortality in this hemodialysis setting.

### Limitations and Strengths

This study has several limitations. First, because ABO blood type was analyzed at the phenotypic level, we were unable to assess underlying ABO gene variants or distinguish between subtypes such as A1 and A2. In addition, as this study relied on medical record data, minor nondifferential misclassification of ABO blood type because of documentation or data-entry errors cannot be completely excluded. Second, as this was an observational study, residual confounding by unmeasured factors cannot be fully excluded despite extensive adjustment and propensity score matching. Such factors may include endothelial function, inflammation beyond baseline C-reactive proteins, residual renal function, vascular calcification, a uremic milieu, blood–membrane interactions, the severity of cardiovascular disease before dialysis initiation, genetic background, and socioeconomic status. Third, as covariates were assessed only at baseline, time-dependent changes in clinical, laboratory parameters, and medication use during follow-up could not be accounted for, which may have influenced the observed associations. Fourth, because the classification of causes of death relied on clinical information across multiple participating centers, some degree of outcome misclassification cannot be completely excluded. Fifth, this study was based on a prevalent hemodialysis cohort rather than an incident cohort. Although dialysis vintage was adjusted for in the multivariable models and stratified analyses by dialysis duration were performed, these approaches cannot fully eliminate the selection bias inherent to prevalent cohort studies, including potential survivor bias. Sixth, the comparison between blood type A and non-A was not prespecified and should therefore be interpreted as exploratory. Furthermore, given the observational design and the potential for residual confounding, the overall findings of this study should also be interpreted as hypothesis-generating rather than causal. Finally, the study population consisted almost exclusively of Japanese patients undergoing hemodialysis. Therefore, the generalizability of our findings to other ethnic groups or patients with end-stage kidney disease treated with modalities other than hemodialysis may be limited. Future studies using incident hemodialysis cohorts across diverse populations, incorporating more detailed genetic characterization and time-updated clinical and laboratory variables, such as dialysis-specific biological markers, will be required to further clarify the mechanisms underlying the observed associations.

Nevertheless, this relatively large, multicenter prospective cohort of 1671 patients undergoing hemodialysis, with 464 documented deaths (278 cardiovascular and 186 noncardiovascular), is, to our knowledge, the first to systematically evaluate the associations between ABO blood type and cause-specific mortality in this high-risk population. The substantial number of events provided adequate statistical power, enabling comprehensive subgroup and multiple sensitivity analyses.

## Conclusion

ABO blood type was associated with all-cause and cardiovascular mortality in patients undergoing hemodialysis, with lower risks observed in those with blood type A, but not with noncardiovascular mortality. These findings suggest that blood type A may be associated with lower cardiovascular mortality in this high-risk population.

## Disclosure

TS has received grants from Astellas Pharma Inc (RS01442), Chugai Pharmaceuticals Co Ltd (AC-1-20150630164707-161208), and Daiichi Sankyo Co Ltd (A16-0602). The other authors declared that they have no competing interests.

## Supplementary Material

Supplementary file (PDF) **Figure S1.** Flow diagram of participant selection. **Table S1.** Sensitivity analyses of the association between ABO blood type (A vs. non-A) and cardiovascular mortality stratified by sudden and nonsudden cardiovascular death. **Table S2.** Baseline clinical characteristics of patients with blood type A and non-A before and after propensity score matching. **Table S3.** Incremental predictive performance of ABO blood type (modeled as 4 categories) for all-cause mortality in Cox proportional hazards models. STROBE Checklist

## Footnotes

Supplementary file (PDF)
**Figure S1.** Flow diagram of participant selection. **Table S1.** Sensitivity analyses of the association between ABO blood type (A vs. non-A) and cardiovascular mortality stratified by sudden and nonsudden cardiovascular death. **Table S2.** Baseline clinical characteristics of patients with blood type A and non-A before and after propensity score matching. **Table S3.** Incremental predictive performance of ABO blood type (modeled as 4 categories) for all-cause mortality in Cox proportional hazards models. STROBE Checklist
