# Supplementary material for: ABO Blood Types and Mortality in Patients Undergoing Hemodialysis
Source: Kidney Int Rep. 2026 Apr 22;11(7):106558. doi: 10.1016/j.ekir.2026.106558 (PMC13200017; doi:10.1016/j.ekir.2026.106558)
Supplement: Supplementary file (PDF) — Figure S1. Flow diagram of participant selection. Table S1. Sensitivity analyses of the association between ABO blood type (A vs. non-A) and cardiovascular mortality stratified by sudden and nonsudden cardiovascular death. Table S2. Baseline clinical characteristics of patients with blood type A and non-A before and after propensity score matching. Table S3. Incremental predictive performance of ABO blood type (modeled as 4 categories) for all-cause mortality in Cox proportional hazards models. STROBE Checklist [file mmc1.pdf]

Supplementary Figure S1. Flow diagram of participant selection

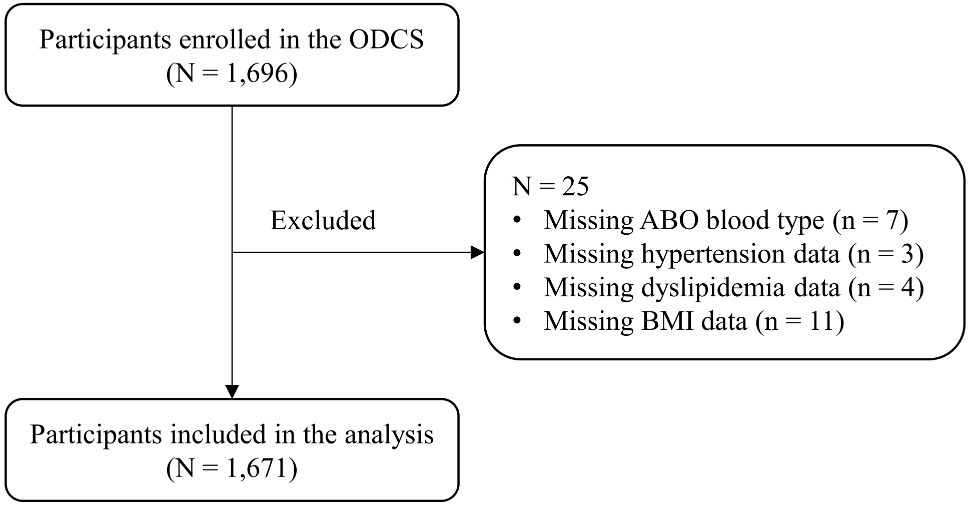

Supplementary Table S1. Sensitivity analyses of the association between ABO blood type (A vs. non-A) and cardiovascular mortality stratified by sudden and non-sudden cardiovascular death

Outcome	Exposure	HR (95% CI)	P value
Sudden cardiovascular death	Blood type A (ref. non-A)	0.851 (0.576–1.256)	0.42
Non-sudden cardiovascular death	Blood type A (ref. non-A)	0.689 (0.489–0.970)	0.033

HRs were estimated using Fine–Gray subdistribution hazard models.

Sudden death was defined as death occurring within 24 hours of symptom onset in the absence of evidence of accident or crime. Non-sudden cardiovascular death included all other cardiovascular causes.

Adjusted using the same covariates as in the primary multivariable Fine–Gray model.

Abbreviations: HR, hazard ratio; CI, confidence interval.

Supplementary Table S2. Baseline clinical characteristics of patients with blood type A and non-A before and after propensity score matching

	Before propensity score matching			After propensity score matching		
	A (*n* = 650)	Non-A (*n* = 1,021)	ASD	A (*n* = 645)	non-A (*n* = 645)	ASD
Age, years	66 (59-74)	68 (61-75)	0.098	66 (59-74)	67 (60-74)	0.006
Female sex, n (%)	232 (35.7%)	387 (37.9%)	0.046	230 (35.7%)	230 (35.7%)	<0.001
BMI, kg/m^2	21.3 (19.0-23.6)	21.1 (19.0-23.6)	0.020	21.3 (19.0-23.6)	21.2 (19.1-23.6)	0.025
Current smoking, n (%)	112 (17.2%)	146 (14.3%)	0.080	109 (16.9%)	107 (16.6%)	0.008
Underlying disease and comorbidities						
DKD, n (%)	269 (41.4%)	414 (40.5%)	0.017	266 (41.2%)	270 (41.9%)	0.013
Hypertension, n (%)	592 (91.1%)	917 (89.8%)	0.043	587 (91.0%)	582 (90.2%)	0.027
Dyslipidemia, n (%)	414 (63.7%)	621 (60.8%)	0.059	409 (63.4%)	412 (63.9%)	0.010
Prior medical history						
CVD, n (%)	246 (37.8%)	387 (37.9%)	0.001	245 (38.0%)	253 (39.2%)	0.025
Dialysis-related parameters						
Dialysis vintage, months	64 (28-133)	64 (29-132)	0.027	65 (28-133)	61 (29-126)	0.022
Laboratory parameters						
Hemoglobin, g/dL	10.7 (10.0-11.3)	10.6 (9.9-11.3)	0.040	10.6 (10.0-11.3)	10.7 (10.0-11.3)	0.001
Albumin, g/dL	3.7 (3.5-3.9)	3.7 (3.5-3.9)	0.095	3.7 (3.5-3.9)	3.7 (3.5-3.9)	0.012
CRP, mg/dL	0.10 (0.05-0.30)	0.10 (0.05-0.33)	0.005	0.10 (0.05-0.30)	0.10 (0.05-0.30)	0.037
Calcium, mg/dL	8.9 (8.5-9.4)	8.9 (8.5-9.5)	0.009	8.9 (8.5-9.4)	8.9 (8.5-9.5)	0.048

Phosphate, mg/dL	5.1 (4.3-5.8)	5.1 (4.3-6.0)	0.012	5.1 (4.3-5.8)	5.1 (4.2-5.9)	0.019
Intact PTH, pg/mL	118 (65-182)	112 (56-192)	0.020	117 (66-182)	112 (54-194)	0.020
Medications						
ESA, n (%)	538 (82.8%)	838 (82.1%)	0.018	533 (82.6%)	540 (83.7%)	0.029
Iron preparations, n (%)	182 (28.0%)	284 (27.8%)	0.004	179 (27.8%)	172 (26.7%)	0.024
VDRA, n (%)	447 (68.8%)	696 (68.2%)	0.013	444 (68.8%)	436 (67.6%)	0.027

Values are expressed as median (interquartile range) for continuous variables and number (percentage) for categorical variables.

Abbreviations: ASD, absolute standardized difference; BMI, body mass index; DKD, diabetic kidney disease; CVD, cardiovascular disease; CRP, C-reactive protein; PTH, parathyroid hormone; ESA, erythropoiesis-stimulating agents; VDRA, vitamin D receptor activators.

Supplementary Table S3. Incremental predictive performance of ABO blood type (modeled as four categories) for all-cause mortality in Cox proportional hazards models

Model	Harrell's C-statistic	ΔC-statistic	LRT (χ^2, df)	P value (LRT)
Base model*	0.749	–	–	–
Base model + ABO (4 groups)	0.751	+0.0025	9.57 (df = 3)	0.023

*Base model covariates: age, sex, dialysis vintage, DKD, history of CVD, current smoking, hypertension, dyslipidemia, hemoglobin, ESA use, iron use, serum calcium, phosphate, intact PTH, VDRA use, BMI, serum albumin, and log CRP.

ABO blood type (A, B, and AB vs O) was entered as a categorical variable with type O as the reference category.

Abbreviations: C, concordance; LRT, likelihood ratio test; df, degrees of freedom; DKD, diabetic kidney disease; CVD, cardiovascular disease; ESA, erythropoiesis-stimulating agents; PTH, parathyroid hormone; VDRA, vitamin D receptor activators; BMI, body mass index; CRP, C-reactive protein.

The STROBE reporting checklist

For checking that observational epidemiology research articles can be understood and used by everyone

How to use this reporting checklist

This reporting checklist allows authors to demonstrate that their manuscripts adhere to the STROBE reporting guideline.

If you have not used a reporting guideline before, read about how and why to use them and check whether STROBE is the most applicable reporting guideline for your work.

Reporting guidelines are most useful when used early in research. When writing a manuscript or application, consider using the full guidance where you'll find explanations and examples for each item.

After writing, demonstrate adherence by completing this checklist:

1. Specify where each item is described (see Note 1).
2. Cite this checklist (See Note 2).
3. Include your completed checklist as a supplement when submitting to a journal so that future readers can use it to find information.

	Item Description	Location (or reason for not reporting)
**Title and abstract**		
1a. Indicate the study's design	Indicate the study's design with a commonly used term in the title or the abstract.	Multicenter prospective cohort study
1b. Abstract	Provide in the abstract an informative and balanced summary of what was done and what was found.	Abstract
**Introduction**		
2. Background / rationale	Explain the scientific background and rationale for the investigation being reported.	Introduction, paragraphs 1–2
3. Objectives	State specific objectives, including any prespecified hypotheses.	Introduction, paragraphs 1–2
**Methods**		
4. Study design	Present key elements of study design early in the paper.	Introduction, last paragraph
5. Setting	Describe the setting, locations, and relevant dates, including periods of recruitment, exposure, follow-up, and data collection.	Methods, Study design and participants

6a. Eligibility criteria	**Cohort study:** Give the eligibility criteria, and the sources and methods of selection of participants. Describe methods of follow-up. **Case-control study:** Give the eligibility criteria, and the sources and methods of case ascertainment and control selection. Give the rationale for the choice of cases and controls. **Cross-sectional study:** Give the eligibility criteria, and the sources and methods of selection of participants.	Methods, Study design and participants
6b. Matching criteria	**Cohort study:** For matched studies, give matching criteria and number of exposed and unexposed. **Case-control study:** For matched studies, give matching criteria and the number of controls per case.	Not applicable (propensity score matching was performed as a sensitivity analysis; see Methods, Statistical analysis)
7. Variables	Clearly define all outcomes, exposures, predictors, potential confounders, and effect modifiers. Give diagnostic criteria, if applicable.	Methods, Outcomes<br>Methods, ABO blood types<br>Methods, Other variables
8. Data sources / measurement	For each variable of interest give sources of data and details of methods of assessment (measurement). Describe comparability of assessment methods if there is more than one group.	Methods, ABO blood types<br>Methods, Other variables
9. Bias	Describe any efforts to address potential sources of bias.	Methods, Statistical analysis
10. Study size	Explain how the study size was arrived at.	Methods, Study design and participants
11. Quantitative variables	Explain how quantitative variables were handled in the analyses. If applicable, describe which groupings were chosen, and why.	Methods, Statistical analysis
12a. Statistical methods	Describe all statistical methods, including those used to control for confounding.	Methods, Statistical analysis (Cox proportional hazards models and Fine–Gray subdistribution hazard models)
12b. Statistical methods – subgroups and interactions	Describe any methods used to examine subgroups and interactions.	Methods, Statistical analysis (subgroup analyses and interaction testing)
12c. Statistical methods – missing data	Explain how missing data were addressed.	Methods, Study design and participants<br>Methods, Statistical analysis<br>Results, Selection of patients
12di. Statistical methods – loss to follow-up	**Cohort study:** If applicable, describe how loss to follow-up was addressed.	Methods, Statistical analysis

12dii. Statistical methods – matching cases and controls	**Case-control study:** If applicable, explain how matching of cases and controls was addressed.	Not applicable
12diii. Statistical methods – sampling strategy	**Cross-sectional study:** If applicable, describe analytical methods taking account of sampling strategy.	Not applicable
12e. Statistical methods – sensitivity analyses	Describe any sensitivity analyses.	Methods, Statistical analysis (seven sensitivity analyses)
**Results**		
13a. Participant numbers	Report the numbers of individuals at each stage of the study—e.g., numbers potentially eligible, examined for eligibility, confirmed eligible, included in the study, completing follow-up, and analysed; Consider use of a flow diagram.	Results, Selection of patients<br>Supplementary Figure S1
13b. Participants – non-participation	Give reasons for non-participation at each stage.	Results, Selection of patients<br>Supplementary Figure S1
13c. Participants – flow diagram	Consider use of a flow diagram.	Supplementary Figure S1
14a. Descriptive data – participant characteristics	Give characteristics of study participants (e.g., demographic, clinical, social) and information on exposures and potential confounders. Present the information in a table.	Results, Clinical characteristics of patients<br>Table 1
14b. Descriptive data – missing data	Indicate the number of participants with missing data for each variable of interest.	Results, Selection of patients
14c. Descriptive data – follow-up time	**Cohort study:** Summarise follow-up time—e.g., average and total amount.	Results, Observed outcomes during the follow-up period
15. Outcome data	**Cohort study:** Report numbers of outcome events or summary measures over time. **Case-control study:** Report numbers in each exposure category, or summary measures of exposure. **Cross-sectional study:** Report numbers of outcome events or summary measures.	Results, Observed outcomes during the follow-up period
16a. Main results	Give unadjusted estimates and, if applicable, confounder-adjusted estimates and their precision (e.g., 95% confidence intervals). Make clear which confounders were adjusted for and why they were included.	Results, ABO blood type and all-cause mortality<br>Results, ABO blood type and cardiovascular mortality<br>Results, ABO blood type and non-cardiovascular mortality<br>Figure 1<br>Figure 2<br>Table 2

		Table 3
16b. Main results – category boundaries	Report category boundaries when continuous variables were categorised.	Not applicable
16c. Main results – risk	If relevant, consider translating estimates of relative risk into absolute risk for a meaningful time period.	Not applicable
17. Other analyses	Report other analyses done—e.g., analyses of subgroups and interactions, and sensitivity analyses.	Results, ABO blood type and cardiovascular mortality<br>Table 4<br>Figure 3<br>Figure 4<br>Supplementary Tables S1<br>Supplementary Tables S2<br>Supplementary Tables S3
**Discussion**		
18. Key results	Summarise key results with reference to study objectives.	Discussion, paragraph 1
19. Limitations	Discuss limitations of the study, taking into account sources of potential bias or imprecision. Discuss both direction and magnitude of any potential bias.	Discussion, Limitations and strengths
20. Interpretation	Give a cautious overall interpretation considering objectives, limitations, multiplicity of analyses, results from similar studies, and other relevant evidence.	Discussion, including interpretation and limitations
21. Generalisability	Discuss the generalisability (external validity) of the study results.	Discussion, geographic differences paragraph<br>Discussion, Limitations and strengths
**Other information**		
22. Funding	Give the source of funding and the role of the funders for the present study and, if applicable, for the original study on which the present article is based.	Funding section
